# Optimization of culture conditions by response surface methodology and unstructured kinetic modeling for bioactive metabolite production by *Nocardiopsis litoralis* VSM-8

**DOI:** 10.1007/s13205-016-0535-2

**Published:** 2016-10-08

**Authors:** Ushakiranmayi Managamuri, Muvva Vijayalakshmi, Sudhakar Poda, V. S. Rama Krishna Ganduri, R. Satish Babu

**Affiliations:** 1 Department of Botany and Microbiology, Acharya Nagarjuna University, Nagarjuna Nagar, Guntur, 52510 Andhra Pradesh India; 2 Department of Biotechnology, Acharya Nagarjuna University, Nagarjuna Nagar, Guntur, 52510 Andhra Pradesh India; 3 Department of Biotechnology, K L University, Vaddeswaram, Guntur, Andhra Pradesh India; 4 Department of Biotechnology, National Institute of Technology, Warangal, Telangana India

**Keywords:** Optimization, Bioactive metabolites, RSM, CCD, Kinetic modeling

## Abstract

Response surface methodology-based central composite design on five variables incubation time, pH, temperature, sucrose concentration, and soya peptone concentration was employed for optimization of the production of bioactive compounds by *Nocardiopsis litoralis* strain VSM 8. The main quadratic effects and interactions of the five variables on the production of bioactive metabolites were investigated. A second-order polynomial model produced a satisfied fit for experimental data with regard to the production of the bioactive metabolites. Regression analysis showed that high *R* ^2^ values of all the five responses are significant and adjusted *R* ^2^ values showed good agreement with the experimental and predicted values. The present model was used to evaluate the direct interaction and quadratic effects to optimize the physico-chemical parameters for the production of bioactive metabolites that inhibit the pathogenic microorganisms measured in terms of zones of inhibition (responses). Mathematical kinetic model development and estimation of kinetic parameters also showed good approximation in terms of model fitting and regression analysis.

## Introduction

Microorganisms dwelling in extreme environments are prolific producers of several bioactive compounds that have evolved due to adaptation of the extreme environmental conditions in terms of metabolic biochemistry. Actinomycetes are well documented for their capacity to produce bioactive metabolites with complex diversity and diverse biological activities. The genes involved in the synthesis of secondary metabolites remain undiscovered due to absence of transcription under conventional laboratory conditions, but mathematical models predict thousands of unexplored secondary metabolites from actinomycete genomes. Actinomycetes are stable, persistent, and active component of the marine microbial communities. Discovery of novel actinobacteria, which operate unusual metabolic pathways, has the ability to produce complex chemical compounds with potential biological activities.

Optimization of physiological parameters and the supplementary nutrition for the biosynthesis of bioactive metabolites can be achieved by the systemic study. The important parameters such as cultural conditions and media constituents are the significant factors influencing the high yield of antimicrobial compounds. Often the production conditions are very similar among the closely related actinomycetes, and hence, optimization of growth and production conditions is very crucial for the maximum production of potential bioactive compounds (Pfefferle et al. 2000; Kiranmayi et al. 2011). Media optimization explores a sequence of phases with specific set of optimal conditions fixed by different methodologies (Shekar et al. 2014).

Traditional one-factor-at-time (OFAT) optimization is laborious and time-consuming, and also misleading conclusions may be drawn, because individual factor interactions are ignored. Hence, establishing the optimized conditions is cumbersome, and therefore, cultural conditions need to be optimized to maximize the production of bioactive compounds with preserved biological activities (Ilaiyaraja et al. 2015). Statistical optimization approach is advantageous than to the traditional practice of changing one variable at a time (Collaa et al. 2016). Response surface methodology (RSM) quantifies the relationship between the controllable input parameters and obtained response. RSM is a powerful statistical experimental approach used in mathematical modeling, and an ideal process for variable standardizing strategy for optimization of the target metabolite production and simultaneously evaluates the relative significance and interactive effects among different variables (Souagui et al. 2015). Central composite design (CCD) is a program of RSM that is embedded with factorial or fractional factorial design with center points that are augmented with a group of “star points” that allows estimation of curvature.

Furthermore, the successful design and operation of fermentation process, in which biochemical transformation occurs in controlled conditions, need careful understanding of complex metabolic reactions. This could be supported by mathematical modeling that describes the process simpler with good representation. Fermentation kinetic models allow the bioengineer to get insight and deep knowledge on the mechanism of synthesis of bioactive metabolites for its yield and productivity from fermentation studies. Furthermore, the evaluation of assumed unstructured models with the experimental data for comparison to find the best model that describes the system. In general, unstructured models consider the cell mass as a whole to explain the biological system and are more effective in elucidating the fermentation profiles of microbial process as for bio products (Rajendran and Thangavelu 2008; Rama Krishna et al. 2016).

Hence, the objectives of this study include the statistical optimization of process parameters for bioactive metabolite production using RSM and to estimate the kinetic parameters of actinomycetes fermentation using *N. litoralis* strain VSM 8 (KT901293).

## Materials and methods

### Isolation and identification of actinomycetes

As a part of our ongoing research on bioactive metabolites, one promising strain with good antimicrobial potential was identified as *N. litoralis* strain VSM 8, isolated from deep sea sediment samples of Bay of Bengal, Visakhapatnam using soil dilution plate technique on actinomycetes isolation agar medium containing 2-g sodium caseinate, 0.1-g l-asparagine, 4-g sodium propionate, 0.5-g dipotassium phosphate, 0.1-g magnesium sulfate, 0.001-g ferrous sulfate, and 5 ml glycerol per 1000 ml distilled water (pH 8). The strain has been deposited in NCBI Genbank with an accession number KT901293. The strain was maintained on Yeast extract Malt Extract Dextrose (YMD) agar medium at 4 °C. An attempt was made in the study to optimize the culture conditions for enhancing the production of bioactive metabolites by the strain using RSM.

### Optimization of screened process parameters for bioactive metabolite production by *N. litoralis* strain VSM 8

RSM applies the statistical and mathematical approach for modeling, designing, and analyzing the engineering problems. It is used to optimize the response surface that influences the various parameters and relationship between the input parameters and the obtained response quantitatively (Parmjit 2008). Relationship between response and independent variables is unknown. Therefore, it is important to execute RSM to find the legitimate practical relation between the responses and the set of independent variables. In this study, the use of RSM to determine the optimum conditions of *N. litoralis* strain VSM 8 for the bioactive metabolite production under a wide range of physical conditions was performed.

To design minimum number of experimental runs, a full factorial central composite face-centered design (CCFD) with five independent variables and their combinations was used to optimize the response with the region of three-dimensional observation spaces. Design Expert software (Version 7.0 State-Ease, Inc., USA) was used to design the experiments for bioactive metabolites production. Using CCD of RSM, the most significant variables (A, B, C, D, and E) at their optimum levels were identified for maximal response in terms of antimicrobial activity of bioactive metabolites measured as zone of inhibition. Five independent variables selected in this study include A-Incubation time, B-pH, C-Temperature, D-Sucrose, and E-Soya peptone. A total of 50 experiments were obtained using following equation that have 2^5^ full factorial CCD for five variables comprising 32 factorial points, 10 axial points, and 8 replicates:

1 $$N = 2^{n} + 2n + n_{\text{c }} \,\,{ = 2}^{ 5} + 2\times 5+ 8= 50,$$

where *N* is total number of experimental runs to be performed, *n* is number of variables (factors), and *n*_c_ is number of replicates at center points.

The central coded value of all the variables was considered as zero. Low and high ranges of all the variables used in RSM and the complete experimental plan with values in actual and coded form are presented in Table 1.

**Table 1:** Experimental range of factors studied using CCD in terms of coded and actual factors

Factors	Symbols	Actual levels of coded factors		
		−1 (low)	0 (middle)	+1 (high)
Time of incubation (days)	*A*	10	11	12
pH	*B*	7	8	9
Temperature (°C)	*C*	25	30	35
Sucrose concentration (%w/v)	*D*	1	2	3
Soya peptone concentration (%w/v)	*E*	0.5	1.0	1.5

### Statistical analysis

The model was statistically analyzed to evaluate the analysis of variance (ANOVA). To analyze the fit and prediction accuracy of the model constructed, correlation coefficients (*R* ^2^), adjusted determination coefficient (Adjusted-*R* ^2^), root mean square error (RMSE), and absolute average deviation (AAD) were carried out between experimental and predicted data. The data obtained were subjected to graphical and regression analysis using Design Expert software. The experimental errors and reproducibility of the data were determined by the central points. To minimize the effect of the uncontrolled factors, the experimental sequence was randomized. The quadratic regression equation was used with each variable to develop an empirical model which correlated the response (bioactive metabolite production) to five variables, as per the following equation (Cui et al. 2016):

2 $$Y = \beta_{0} + \mathop \sum \limits_{i = 1}^{n} \beta_{i} X_{i} + \left( {\mathop \sum \limits_{i = 1}^{n} \beta_{ii} X_{i} } \right)^{2} + \mathop \sum \limits_{i = 1}^{n - 1} \mathop \sum \limits_{j = i + 1}^{n} \beta_{ij} X_{i} X_{j} ,$$

where *Y* is predicted response, *β*_0_ is intercept coefficient, *β*_*i*_ is linear coefficient, *β*_*ij*_ is interaction coefficients, *β*_*ii*_ is quadratic coefficients, and *X*_*i*_ and *X*_*j*_ are coded values of the five additive variables.

### Unstructured kinetic modeling of *N. litoralis* strain VSM 8

The growth of halophilic marine actinomycete with limiting carbon substrates influences the bioactive metabolite production. Basic mathematical and unstructured kinetic models quantitatively describe the substrate utilization and growth-associated production formation kinetics in a batch system, and the similar equations were also developed by many researchers (Mohammad et al. 1995; Cheng et al. 2010; Li et al. 2015). Models of logistic and Luedeking–Piret were used to simulate the cell growth and bioactive metabolite production of *N. litoralis* strain VSM 8 (KT901293). The data acquired from the models were used to calculate the specific cell growth rate (*µ*_max_), day^−1^, specific production rate of bioactive metabolite, day^−1^.

Under optimal growth conditions and no effects of substrate and product inhibition, growth kinetic model of *N. litoralis* strain VSM 8 (KT901293) (*X*) (as per Malthus’s law), in a batch fermentation, is best described as logistic function (Leroy and de Vuyst 1999):

3 $$\frac{{{\text{d}}X}}{{{\text{d}}t}} = \mu_{ \hbox{max} } X\left( {1 - \frac{X}{{X_{\text{m}} }}} \right).$$

On integration, the above equation gives the logistic (L)-type model equation that relates hyperbolic growth of cell:

4 $$X(t) = \frac{{X_{0} e^{{\mu_{ \hbox{max} } t}} }}{{1 - \frac{{X_{0} }}{{X_{\text{m}} }}(1 - e^{{\mu_{ \hbox{max} } t}} )}},$$

where *X* is biomass concentration, g/l, *µ*_max_ is the maximum specific cell growth rate, day^−1^, and *X*_m_ is the maximum biomass concentration, g/l.

Bioactive metabolite production can be obtained from growth limiting substrate (optimized media constituents) and the substrate utilization kinetics can be taken from Modified Leudeking–Piret (MLP) equation:

5 $$- \frac{{{\text{d}}S}}{{{\text{d}}t}} = r_{\text{S}} = \gamma \left( {\frac{{{\text{d}}X}}{{{\text{d}}t}}} \right) + \eta X.$$

On integration, the above equation results logistic incorporated modified Leudeking–Piret (LIMLP) equation:

6 $$S(t) = S_{0} - \gamma \left[ {\frac{{X_{0} e^{{\mu_{ \hbox{max} } t}} }}{{1 - \left( {\frac{{X_{0} }}{{X_{\text{m}} }}} \right)\left( {1 - e^{{\mu_{ \hbox{max} } t}} } \right)}} - X_{0} } \right] + \frac{{\eta X_{\text{m}} }}{{\mu_{ \hbox{max} } }}{ \ln }\left[ {1 - \left( {\frac{{X_{0} }}{{X_{\text{m}} }}} \right)\left( {1 - e^{{\mu_{ \hbox{max} } t}} } \right)} \right].$$

Constant of non-growth-associated substrate consumption, *η*, in above equation can be calculated from stationary phase data(where $\frac{{ - {\text{d}}S}}{{{\text{d}}t}} = 0$):

7 $$\eta = \frac{{ - \left( {\frac{{{\text{d}}S}}{{{\text{d}}t}}} \right)_{\text{stationary phase}} }}{{X_{ \hbox{max} } }}.$$

Significant bioactive metabolite (product) formation occurs in late-logarithmic phase of cell growth and bioactive metabolite formation kinetics follows Leudeking–Piret equation (Luedeking and Piret 2000), as:

8 $$\frac{{{\text{d}}P}}{{{\text{d}}t}} = \alpha \frac{{{\text{d}}X}}{{{\text{d}}t}} + \beta X.$$

Logistic incorporated Leudeking–Piret (LILP) equation derived from integration of the above equation results:

9 $$P(t) = P_{0} + \alpha \left[ {\frac{{X_{0} e^{{\mu_{ \hbox{max} } t}} }}{{1 - \left( {\frac{{X_{0} }}{{X_{\text{m}} }}} \right)\left( {1 - e^{{\mu_{ \hbox{max} } t}} } \right)}} - X_{0} } \right] + \frac{{\beta X_{\text{m}} }}{\mu }{ \ln }\left[ {1 - \left( {\frac{{X_{0} }}{{X_{\text{m}} }}} \right)\left( {1 - e^{{\mu_{ \hbox{max} } t}} } \right)} \right].$$

Non-growth-associated product formation constant, *β*, can be determined from stationary phase data (where $\frac{{{\text{d}}X}}{{{\text{d}}t}} = 0$):

10 $$\beta = \frac{{\left( {\frac{{{\text{d}}P}}{{{\text{d}}t}}} \right)_{\text{stationary phase}} }}{{X_{ \hbox{max} } }}.$$

Experimental data obtained from batch shake-flask fermentations was used to simulate using Eqs. (4), (6), and (9).

## Results

### RSM modeling

Influence of different physico-chemical parameters on bioactive metabolite production and their effect on the response (measured as inhibition zones) were investigated and optimized as per the model designed by CCD of RSM. The effect of independent variable optimization on the responses was identified by complete five factors and three-level factorial experiment designs with eight replications of central point and ten axial points and thirty-two factorial points for bioactive metabolite production by the strain. The maximum production of the bioactive compound and its effect on the responses (inhibition of growth of the pathogenic microorganisms by the bioactive compound produced by *N. litoralis* strain VSM 8 is represented in mm) was experimentally found to be 21 mm (*Staphylococcus aureus*), 20 mm (*Bacillus subtilis*), 22.9 mm (*X. campestris*), 19.9 mm (*Pseudomonas aeruginosa*), and 17.9 mm (*Candida albicans*) that was obtained from cultural conditions of the strain grown in a medium containing 2 % sucrose, 1 % soya peptone with pH 8 incubated at 25 °C for 11 days.

To determine whether the model would give misleading or approximate results, the experimental data are subjected to model adequacy. Linear, interactive, quadratic, and cubic models were fitted to the experimental data to determine the actual relationship between the response and the variables selected for the study. The suggested sequential model sum of squares and lack of fit tests (showing degrees of freedom; mean square, *F* value, *p* value), model summary statistics (showing standard deviation, *R* ^2^, adjusted *R* ^2^ and predicted *R* ^2^) for the best outcome quadratic model (Bipasha et al. 2015), for all the five responses, are given in Table 2.

**Table 2:** Sequential model fitting for all the responses (in terms of inhibition zone produced by bioactive metabolite)

Model parameter	*S. aureus*	*B. subtilis*	*X. campestris*	*P. aeruginosa*	*C. albicans*
Sequential model sum of squares—quadratic vs 2FI (suggested)					
Sum of squares	85.83	59.22	237.14	94.56	26.82
*df*	5	5	5	5	5
Mean square	11.17	11.84	47.43	18.91	5.36
*F* value	2157.78	240.13	982.19	317.08	590.81
*p* value (Prob > *F*)	<0.0001	<0.0001	<0.0001	<0.0001	<0.0001
Lack of fit tests—quadratic (suggested)					
Sum of squares	0.15	0.71	1.40	1.73	0.26
*df*	20	20	20	20	25
Mean square	7.535E−003	0.036	0.0070	0.086	1.08
*F* value	0.85	0.44	–	–	–
*p* value (Prob > *F*)	0.6410	0.9373	–	–	–
Model summary statistics—quadratic (suggested)					
Std. Dev.	0.089	0.22	0.22	0.24	0.095
*R* ^2^	0.9975	0.9797	0.9944	0.9824	0.9925
Adjusted *R* ^2^	0.9957	0.9657	0.9906	0.9702	0.9873
Predicted *R* ^2^	0.9931	0.9503	0.9811	0.9551	0.9781

*df* degrees of freedom

As per the sequential model sum of squares of all the five responses, the quadratic model was significant with (*p* value <0.0001) for responses *Staphylococcus aureus*, *Bacillus subtilis*, *Xanthomonas campestris*, *Pseudomonas aeruginosa,* and *Candida albicans* (inhibition of the growth was represented in mm). The lack of fit test values of quadratic models identified earlier as the accepted model for all the responses was found to be insignificant lack of fit which indicate that the model is highly significant. The second-order quadratic model equations and regression coefficients (%) of all responses (Urailuck et al. 2015) were given in Table 3.

**Table 3:** The second-order quadratic model equations and regression coefficients (%) of responses

Response	Second-order quadratic model equation (in coded factors)	Regression coefficient (%)	
		*R* ^2^	$R_{adj.}^{2}$
*Staphylococcus aureus*	*Y* = 20.924 + 0.0957321 × *A* + −0.195509 × *B* + 0.0896271 × *C* + 0.189804 × *D* + 0.0898044 × *E* + 0.00453468 × *AB* + −0.00453468 × *AC* + −0.00591517 × *AD* + −0.00591517 × *AE* + 0.0110212 × *BC* + 0.0108328 × *BD* + 0.0108328 × *BE* + −0.0108328 × *CD* + −0.0108328 × *CE* + −0.0110212 × *DE* + −0.54795 × *A* ^2^ + −0.14795 × *B* ^2^ + −0.34795 × *C* ^2^ + −1.04795 × *D* ^2^ + −0.94795 × *E* ^2^	99.75	99.57
*Bacillus subtilis*	*Y* = 20.3477 + 0.127445 × *A* + 0.145469 × *B* + 0.313355 × *C* + 0.167851 × *D* + 0.285498 × *E* + 0.0333395 × *AB* + 0.0291605 × *AC* + −0.00675795 × *AD* + −0.00675795 × *AE* + 0.0483105 × *BC* + 0.0279083 × *BD* + 0.0279083 × *BE* + −0.0154083 × *CD* + −0.0154083 × *CE* + 0.0141895 × *DE* + −0.545489 × *A* ^2^ + −0.545489 × *B* ^2^ + −0.945489 × *C* ^2^ + −0.145489 × *D* ^2^ + −0.345489 × *E* ^2^	97.97	96.57
*Xanthomonas campestris*	*Y* = 22.7623 + 0.3 × *A* + 0.10183 × *B* + 0.0540524 × *C* + 0.3 × *D* + −0.3 × *E* + 1.80021*E*−015 × *AB* + 2.57419*E*−015 × *AC* + 0.00777721 × *AD* + 0.00777721 × *AE* + 0.0019443 × *BC* + 1.8162*E*−015 × *BD* + 2.3339*E*−015 × *BE* + 2.2161*E−*015 × *CD* + 2.35407*E−*015 × *CE* + −0.0019443 × *DE* + −0.924567 × *A* ^2^ + −1.12457 × *B* ^2^ + −0.374567 × *C* ^2^ + −1.12457 × *D* ^2^ + −1.52457 × *E* ^2^	99.44	99.06
*Pseudomonas aeruginosa*	*Y* = 19.5911 + 5.58251*E−*016 × *A* + 0.000670037 × *B* + 0.09933 × *C* + 5.20199*E−*016 × *D* + −0.1*E + −6.15544*E−*016 × *AB* + −8.10404*E−*016 × *AC* + 0.00284766 × *AD* + 0.00284766 × *AE* + 0.000711914 × *BC* + −5.92518*E−*016 × *BD* + −3.0522*E−*016 × *BE* + −6.59243*E−*016 × *CD* + −1.44794*E−*015 × *CE* + −0.000711914 × *DE* + −0.682226 × *A* ^2^ + −0.682226 × *B* ^2^ + −0.582226 × *C* ^2^ + −0.482226 × *D* ^2^ + −0.782226 × *E* ^2^	98.24	97.02
*Candida albicans*	*Y* = 17.8007 + −0.00142264 × *A* + −0.208914 × *B* + 0.100091 × *C* + 0.000523031 × *D* + 0.409347 × *E* + 0.00151156 × *AB* + −0.00151156 × *AC* + −0.000385869 × *AD* + −0.000385869 × *AE* + −9.64674*E−*005 × *BC* + −0.00055572 × *BD* + −0.00055572 × *BE* + 0.00055572 × *CD* + 0.00055572 × *CE* + 9.64674*E−*005 × *DE* + −0.501358 × *A* ^2^ + −0.151358 × *B* ^2^ + −0.401358 × *C* ^2^ + −0.301358 × *D* ^2^ + −0.351358 × *E* ^2^	99.25	98.73

*Y* response, *A* incubation time (days), *B* pH, *C* temperature (°C), *D* sucrose concentration (%w/v), *E* soya peptone concentration (%w/v)

The model was found to be statistically significant with confidence level of 99.9 % since the Prob > *F* value of the model for all the five responses was found to be <0.00001 and *F* values of the model for all the five responses was found to be <0.00001 and *F* values of the model for five responses 569.25, 69.91, 258.49, 80.83, and 190.97 (in terms of inhibition of growth of the pathogenic microorganisms, viz., *Staphylococcus aureus*, *Bacillus subtilis*, *Xanthomonas campestris*, *Pseudomonas aeruginosa*, *Candida* albicans, respectively, by the bioactive compound produced by VSM 8 is represented in mm) implies that the model is significant. Model terms are said to be significant if the values of *p* (Prob < *F*) is less than 0.0500. ANOVA reveals that most of the significant factors affecting the growth inhibition of pathogenic microorganisms (five responses) by the bioactive compounds produced by VSM 8 for the variables: incubation time (days), pH, temperature, concentration of sucrose, and concentration of soya peptone. Tables 4, 5, 6, 7 and 8 show the ANOVA results obtained from statistical optimization.

**Table 4:** ANOVA for *Staphylococcus aureus* response surface quadratic model

Source	Sum of squares	*df* ^a^	Mean square	*F* value	*P* value Prob > *F*	
Model	90.57	20	4.53	569.25	<0.0001	Significant
*A*-time of incubation	0.28	1	0.28	35.02	<0.0001	
*B*-pH	1.26	1	1.26	157.88	<0.0001	
*C*-temperature	0.26	1	0.26	33.18	<0.0001	
*D*-Sucrose	1.16	1	1.16	145.35	<0.0001	
*E*-Soya Peptone	0.26	1	0.26	32.54	<0.0001	
*AB*	5.844E−004	1	5.844E−004	0.073	0.7883	
*AC*	5.844E−004	1	5.844E−004	0.073	0.7883	
*AD*	1.027E−003	1	1.027E−003	0.13	0.7219	
*AE*	1.027E−003	1	1.027E−003	0.13	0.7219	
*BC*	3.749E−003	1	3.749E−003	0.47	0.4979	
*BD*	3.533E−003	1	3.533E−003	0.44	0.5104	
*BE*	3.533E−003	1	3.533E−003	0.44	0.5104	
*CD*	3.533E−003	1	3.533E−003	0.44	0.5104	
*CE*	3.533E−003	1	3.533E−003	0.44	0.5104	
*DE*	3.749E−003	1	3.749E−003	0.47	0.4979	
*A* ^2^	0.74	1	0.74	93.34	<0.0001	
*B* ^2^	0.054	1	0.054	6.80	0.0142	
*C* ^2^	0.30	1	0.30	37.64	<0.0001	
*D* ^2^	2.72	1	2.72	341.39	<0.0001	
*E* ^2^	2.22	1	2.22	279.34	<0.0001	

^a^Degrees of freedom

**Table 5:** ANOVA for *Bacillus subtilis* response surface quadratic model

Source	Sum of squares	*df* ^a^	Mean square	*F* value	*P* value Prob > *F*	
Model	68.97	20	3.45	69.91	<0.0001	Significant
*A*-time of incubation	0.49	1	0.49	10.01	0.0036	
*B*-pH	0.70	1	0.70	14.10	0.0008	
*C*-temperature	3.23	1	3.23	65.41	<0.0001	
*D*-Sucrose	0.90	1	0.90	18.33	0.0002	
*E*-Soya peptone	2.62	1	2.62	53.04	<0.0001	
*AB*	0.0321	1	0.032	0.64	0.4301	
*AC*	0.024	1	0.024	0.49	0.4895	
*AD*	1.341E−003	1	1.341E−003	0.027	0.8702	
*AE*	1.341E−003	1	1.341E−003	0.027	0.8702	
*BC*	0.072	1	0.072	1.46	0.2366	
*BD*	0.023	1	0.023	0.48	0.4960	
*BE*	0.023	1	0.023	0.48	0.4960	
*CD*	7.147E−003	1	7.417E−003	0.14	0.7062	
*CE*	7.147E−003	1	7.147E−003	0.14	0.7062	
*DE*	6.214E−003	1	6.214E−003	0.13	0.7252	
*A* ^2^	0.74	1	0.74	14.92	0.0006	
*B*	0.74	1	0.74	14.92	0.0006	
*C* ^2^	2.21	1	2.21	44.82	<0.0001	
*D* ^2^	0.052	1	0.052	1.06	0.3114	
*E* ^2^	0.30	1	0.30	5.98	0.0207	

^a^Degrees of freedom

**Table 6:** ANOVA for *Xanthomonas campestris* response surface quadratic model

Source	Sum of squares	*df* ^a^	Mean square	*F* value	*P* value Prob > *F*	
Model	249.64	20	12.48	258.49	<0.0001	Significant
*A*-time of incubation	2.74	1	22.74	56.65	<0.0001	
*B*-pH	0.34	1	0.34	7.06	0.0127	
*C*-temperature	0.096	1	0.096	1.99	0.1692	
*D*-Sucrose	2.89	1	2.89	59.82	<0.0001	
*E*-Soya peptone	2.89	1	2.89	59.82	<0.0001	
*AB*	0.000	1	0.000	0.000	1.0000	
*AC*	0.000	1	0.000	0.000	1.0000	
*AD*	1.776E−003	1	1.776E−003	0.037	0.8493	
*AE*	1.776E−003	1	1.776E−003	0.37	0.8493	
*BC*	1.167E−003	1	1.167E−003	2.416E−003	0.9611	
*BD*	0.000	1	0.000	0.000	1.0000	
*BE*	0.000	1	0.000	0.000	1.0000	
*CD*	0.000	1	0.000	0.000	1.0000	
*CE*	0.000	1	0.000	0.000	1.0000	
*DE*	1.167E−003	1	1.167E−003	2.416E−003	0.9611	
*A* ^2^	2.11	1	2.11	43.78	<0.0001	
*B* ^2^	3.13	1	3.13	64.77	<0.0001	
*C* ^2^	0.35	1	0.35	7.19	0.0120	
*D* ^2^	3.13	1	3.13	64.77	<0.0001	
*E* ^2^	5.75	1	5.75	119.03	<0.0001	

^a^Degrees of freedom

**Table 7:** ANOVA for *Pseudomonas aeruginosa* response surface quadratic model

Source	Sum of squares	*df* ^a^	Mean square	*F* value	*P* value Prob > *F*	
Model	96.43	20	4.82	80.83	<0.0001	Significant
*A*-time of incubation	0.000	1	0.000		1.0000	
*B*-pH	1.475E−005	1	1.475E−005	2.473E−004	0.9876	
*C*-temperature	0.32	1	0.32	5.44	0.0269	
*D*-Sucrose	0.000	1	0.000	0.000	1.0000	
*E*-Soya peptone	0.32	1	0.32	5.38	0.0276	
*AB*	0.000	1	0.000	0.000	1.0000	
*AC*	0.000	1	0.000	0.000	1.0000	
*AD*	2.381E−004	1	2.381E−004	3.992E−003	0.9501	
*AE*	2.381E−004	1	2.381E−004	3.992E−003	0.9501	
*BC*	1.564E−005	1	1.564E−005	2.622E−004	0.9872	
*BD*	0.000	1	0.000	0.000	1.0000	
*BE*	0.000	1	0.000	0.000	1.0000	
*CD*	0.000	1	0.000	0.000	1.0000	
*CE*	0.000	1	0.000	0.000	1.0000	
*DE*	1.564E−005	1	1.564E−005	2.622E−004	0.9872	
*A* ^2^	1.15	1	1.15	19.30	0.0001	
*B* ^2^	1.15	1	1.15	19.30	0.0001	
*C* ^2^	0.84	1	0.84	14.05	0.0008	
*D* ^2^	0.58	1	0.58	9.64	0.0042	
*E* ^2^	1.15	1	1.15	25.37	<0.0001	

^a^Degrees of freedom

**Table 8:** ANOVA for *Candida albicans* response surface quadratic model

Source	Sum of squares	*df* ^a^	Mean square	*F* value	*P* value Prob > *F*	
Model	34.68	20	1.73	190.97	<0.0001	Significant
*A*-time of incubation	6.152E−005	1	6.152E−005	6.776E−003	0.9350	
*B*-pH	1.43	1	1.43	157.96	<0.0001	
*C*-temperature	0.33	1	0.33	36.26	<0.0001	
*D*-Sucrose	8.781E−006	1	8.781E−006	9.671E−004	0.9754	
*E*-Soya peptone	5.38	1	5.38	592.38	<0.0001	
*AB*	6.493E−005	1	6.493E−005	7.152E−003	0.9332	
*AC*	6.493E−005	1	6.493E−005	7.152E−003	0.9332	
*AD*	4.372E−005	1	4.372E−005	4815E−004	0.9826	
*AE*	4.372E−005	1	4.372E−005	4815E−004	0.9826	
*BC*	2.872E−007	1	2.872E−007	3.163E−005	0.9956	
*BD*	9.297E−006	1	9.297E−006	1.024E−003	0.9747	
*BE*	9.297E−006	1	9.297E−006	1.024E−003	0.9747	
*CD*	9.297E−006	1	9.297E−006	1.024E−003	0.9747	
*CE*	9.297E−006	1	9.297E−006	1.024E−003	0.9747	
*DE*	2.872E−007	1	2.872E−007	3.163E−005	0.9956	
*A* ^2^	0.62	1	0.62	68.47	<0.0001	
*B* ^2^	0.057	1	0.057	6.24	0.0184	
*C* ^2^	0.40	1	0.40	43.88	<0.0001	
*D* ^2^	0.22	1	0.22	24.74	<0.0001	
*E* ^2^	0.31	1	0.31	33.63	<0.0001	

^a^Degrees of freedom

Regression analysis indicated that the coefficient of determination (*R* ^*2*^) values of the five responses and high value of *R* ^*2*^ indicate that the full quadratic model equation was capable of representing that the system under a given experimental domain is significant and adjusted coefficient of determination ($R_{adj.}^{2}$) values also indicated good agreement between experimental and the predicted values. Degree of precision is indicated by the coefficient variation (CV) with which the experiment is compared. Experimental reliability is poor if the CV value is high. The CV (%) values of the five responses in this study were found to be 0.47, 1.19, 1.14, 1.4, and 0.57 which denote that the performed experiment is reliable. The present model was used to evaluate the direct interaction and quadratic effects to optimize the physico-chemical parameters for the production and bioactive metabolites that inhibit the pathogenic microorganisms (responses). Central composite factor experimental design along with experimental and predicted values is shown in Table 9.

**Table 9:** Central composite factor experimental design along with experimental and predicted values

Run	*A*-time (days)	*B*-pH	*C*-temperature (°C)	*D*-[Sucrose] (%w/v)	*E*-[Soya peptone] (%w/v)	*S. aureus*		*B. subtilis*		*X. campestris*		*P. aeruginosa*		*C. albicans*	
						Actual	RSM	Actual	RSM	Actual	RSM	Actual	RSM	Actual	RSM
1	11.00	7.00	25.00	2.00	1.00	17.60	17.60	16.80	16.92	17.20	17.20	16.40	16.40	15.80	15.80
2	10.00	9.00	30.00	1.00	0.50	17.80	17.82	17.00	17.07	17.80	17.80	16.40	16.40	15.80	15.80
3	11.00	8.00	25.00	2.00	1.50	17.20	17.15	17.00	16.93	17.40	17.40	16.40	16.40	15.40	15.40
4	11.00	8.00	25.00	2.00	1.00	17.40	17.38	17.20	17.22	18.00	18.00	16.40	16.40	15.40	15.40
5	10.00	9.00	20.00	1.00	1.50	17.80	17.81	17.40	17.45	17.40	17.40	16.60	16.60	16.00	16.00
6	10.00	9.00	20.00	1.00	0.50	17.80	17.81	17.40	17.45	17.40	17.40	16.60	16.60	16.00	16.00
7	12.00	9.00	20.00	1.00	1.50	17.40	17.40	17.60	17.33	17.60	17.60	16.60	16.60	15.60	15.60
8	11.00	8.00	25.00	1.00	1.00	17.60	17.62	18.20	18.07	18.20	18.20	16.60	16.60	15.60	15.60
9	12.00	7.00	20.00	1.00	1.50	18.00	18.02	17.20	17.21	17.80	17.80	16.40	16.40	15.80	15.80
10	11.00	9.00	25.00	2.00	1.00	18.00	18.21	17.40	17.34	18.40	18.40	16.40	16.40	15.80	15.80
11	12.00	9.00	30.00	1.00	0.50	17.60	17.61	17.40	17.34	18.00	18.00	16.40	16.40	15.40	15.40
12	11.00	8.00	25.00	2.00	1.00	17.80	17.81	17.60	17.60	18.60	18.60	16.40	16.40	15.40	15.40
13	10.00	7.00	20.00	3.00	0.50	18.20	18.18	17.80	17.68	18.00	18.00	16.60	16.60	16.00	16.00
14	10.00	7.00	20.00	1.00	0.50	18.40	18.35	18.00	17.93	18.60	18.60	16.60	16.60	16.00	16.00
15	12.00	9.00	30.00	3.00	0.50	17.80	17.82	18.00	18.01	18.20	18.20	16.60	16.60	15.60	15.60
16	12.00	7.00	30.00	1.00	1.50	18.00	18.01	18.20	18.39	18.80	18.80	16.60	16.60	15.60	15.60
17	12.00	8.00	25.00	2.00	1.00	17.80	17.82	17.40	17.45	16.60	16.60	16.20	16.20	16.60	16.60
18	10.00	7.00	20.00	3.00	1.50	18.00	18.01	17.60	17.58	17.20	17.20	16.20	16.20	16.60	16.60
19	11.00	8.00	25.00	2.00	1.00	17.40	17.41	17.60	17.58	16.80	16.80	16.20	16.20	16.20	16.20
20	11.00	8.00	20.00	2.00	1.00	17.60	17.61	17.80	17.84	17.40	17.40	16.20	16.20	16.20	16.20
21	10.00	9.00	30.00	1.00	1.50	18.00	17.98	18.00	17.92	16.80	16.80	16.40	16.40	16.80	16.80
22	10.00	8.00	25.00	2.00	1.00	18.20	18.15	18.20	18.17	17.40	17.40	16.40	16.40	16.80	16.80
23	10.00	9.00	20.00	3.00	1.50	17.60	17.62	18.20	18.24	17.00	17.00	16.40	16.40	16.40	16.40
24	12.00	7.00	20.00	3.00	1.50	17.80	17.81	18.40	18.62	17.60	17.60	16.40	16.40	16.40	16.40
25	12.00	9.00	20.00	3.00	0.50	18.20	18.18	17.80	17.80	17.20	17.20	16.20	16.20	16.60	16.60
26	10.00	9.00	30.00	3.00	0.50	18.40	18.15	18.00	17.90	17.80	17.80	16.20	16.20	16.60	16.60
27	12.00	9.00	20.00	3.00	1.50	17.80	17.82	18.00	18.04	17.40	17.40	16.20	16.20	16.20	16.20
28	10.00	7.00	30.00	1.00	1.50	18.00	18.00	18.20	18.28	18.00	18.00	16.20	16.20	16.20	16.20
29	12.00	7.00	30.00	3.00	0.50	18.60	18.46	18.00	18.43	18.00	18.00	16.40	16.40	16.80	1.80
30	12.00	7.00	30.00	3.00	1.50	18.20	18.46	18.60	18.43	18.00	18.00	16.40	16.40	16.80	16.80
31	11.00	8.00	25.00	2.00	1.00	18.00	17.98	18.60	18.64	17.60	17.60	16.40	16.40	16.40	16.40
32	12.00	7.00	30.00	3.00	1.50	18.20	18.15	19.40	19.00	18.20	18.20	16.40	16.40	16.40	16.40
33	10.00	7.00	30.00	1.00	0.50	20.20	20.20	19.80	19.67	21.40	21.40	18.60	18.60	17.20	17.20
34	10.00	9.00	30.00	3.00	1.50	20.40	20.39	20.00	19.93	22.00	22.00	18.60	18.60	17.20	17.20
35	11.00	8.00	25.00	2.00	1.00	20.80	20.79	19.80	19.66	21.40	21.40	18.60	18.60	17.60	17.60
36	12.00	9.00	30.00	3.00	1.50	20.40	20.41	20.00	19.95	21.60	21.60	18.60	18.60	17.20	17.20
37	11.00	8.00	25.00	2.00	0.50	20.40	20.41	19.20	19.09	21.40	21.40	18.60	18.60	17.20	17.20
38	12.00	9.00	20.00	1.00	0.50	20.60	20.59	19.80	19.72	21.60	21.60	18.80	18.80	17.40	17.40
39	11.00	8.00	30.00	2.00	1.00	19.60	19.61	20.40	20.03	21.20	21.20	18.80	18.80	17.40	17.40
40	12.00	9.00	30.00	1.00	1.50	20.00	19.99	20.20	20.37	21.80	21.80	18.80	18.80	17.40	17.40
41	11.00	8.00	25.00	3.00	1.00	19.80	19.81	19.80	19.72	21.40	21.40	18.60	18.60	16.80	16.80
42	11.00.	8.00	25.00	2.00	1.00	20.00	19.99	20.40	20.29	20.80	20.80	18.40	18.40	17.60	17.60
43	10.00	9.00	20.00	3.00	0.50	20.80	20.80	20.40	20.35	22.40	22.40	19.20	19.20	17.60	17.60
44	10.00	7.00	30.00	1.00	0.50	20.80	20.80	19.80	20.35	22.40	22.40	19.20	19.20	17.60	17.60
45	12.00	7.00	20.00	1.00	0.50	20.80	20.80	20.40	20.35	22.40	22.40	19.20	19.20	17.60	17.60
46	10.00	7.00	30.00	3.00	0.50	20.80	20.80	20.40	20.35	22.40	22.40	19.20	19.20	17.60	17.60
47	12.00	7.00	20.00	3.00	0.50	20.80	20.80	19.80	20.35	22.40	22.40	19.20	19.20	17.60	17.60
48	11.00	8.00	25.00	2.00	1.00	20.80	20.80	20.40	20.35	22.40	22.40	19.20	19.20	17.60	17.60
49	11.00	8.00	25.00	2.00	1.00	20.80	20.80	20.40	20.35	22.40	22.40	19.20	19.20	17.60	17.60
50	10.00	7.00	20.00	1.00	1.50	20.80	20.80	20.40	20.35	22.40	22.40	19.20	19.20	17.60	17.60

### Quadratic and interactive effects of bioactive metabolite production and its effect on the responses

**Fig. 1:**
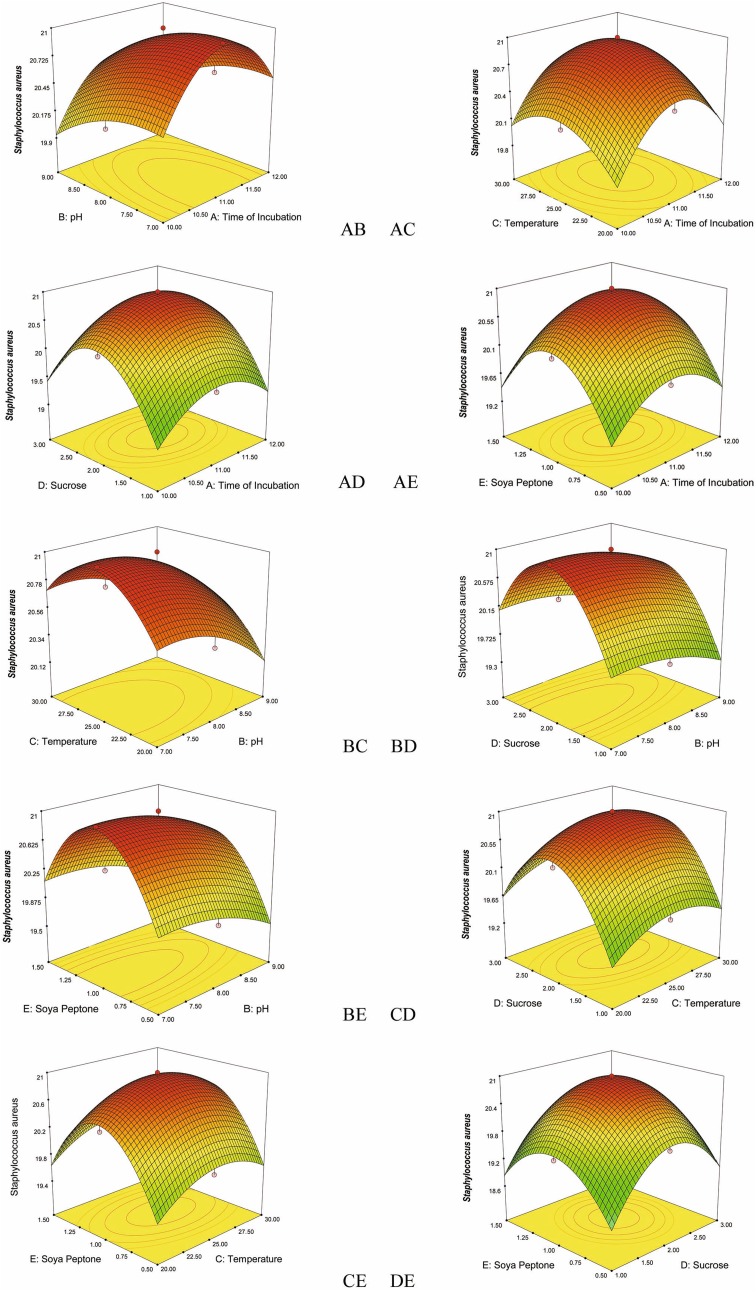
Response surface plots consisting of three-dimensional views and contours showing interactive effects of selective variables on zone of inhibition (mm) of the bioactive compound production by VSM 8 against *Staphylococcus aureus*: (*AB*) time of incubation and pH, (*AC*) time of incubation and temperature, (*AD*) time of incubation and concentration of sucrose, (*AE*) time of incubation and concentration of soya peptone, (*BC*) pH and temperature, (*BD*) pH and concentration of sucrose, (*BE*) pH and concentration of soya peptone, (*CD*) temperature and concentration of sucrose, (*CE*) temperature and concentration of soya peptone, (*DE*) concentration of sucrose and concentration of soya peptone

**Fig. 2:**
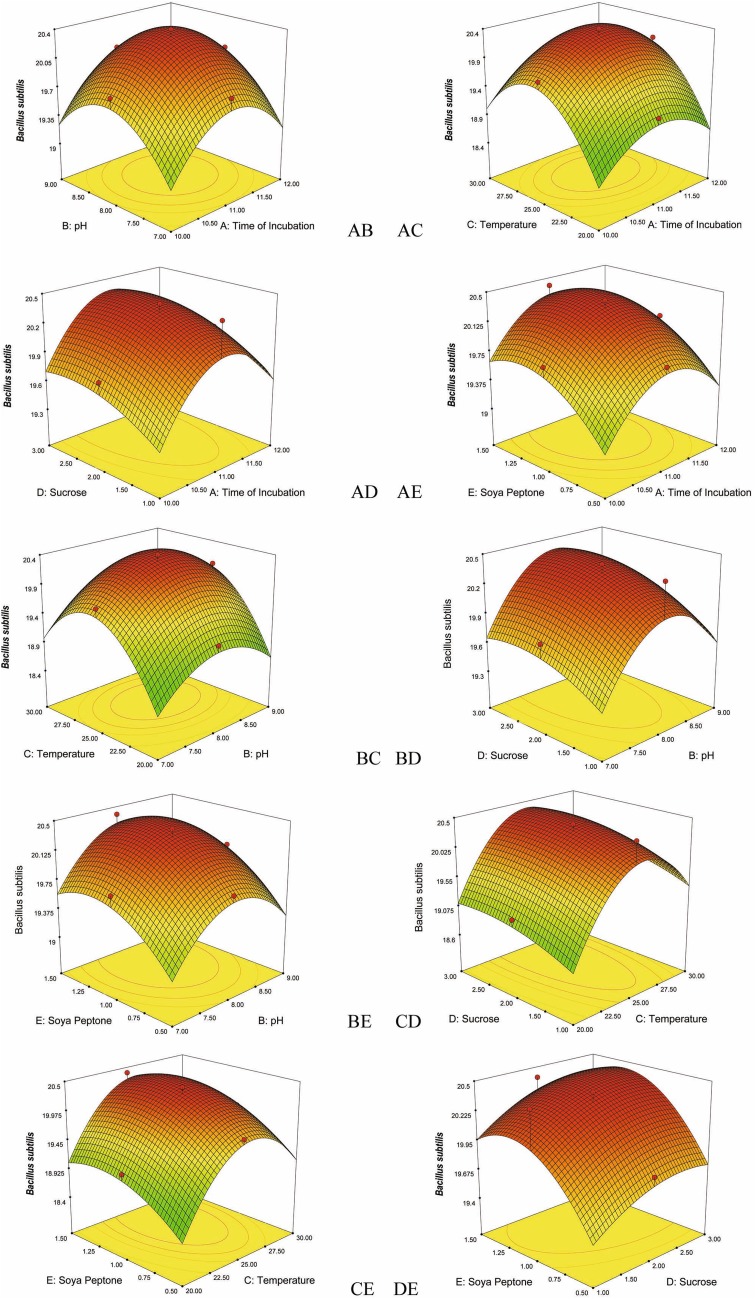
Response surface plots consisting three-dimensional views and contours showing interactive effects of selective variables on zone of inhibition (mm) of the bioactive compound production by VSM 8 against *Bacillus subtilis*: (*AB*) time of incubation and pH, (*AC*) time of incubation and temperature, (*AD*) time of incubation and concentration of sucrose, (*AE*) time of incubation and concentration of soya peptone, (*BC*) pH and temperature, (*BD*) pH and concentration of sucrose, (*BE*) pH and concentration of soya peptone, (*CD*) temperature and concentration of sucrose, (*CE*) temperature and concentration of soya peptone, (*DE*) concentration of sucrose and concentration of soya peptone

**Fig. 3:**
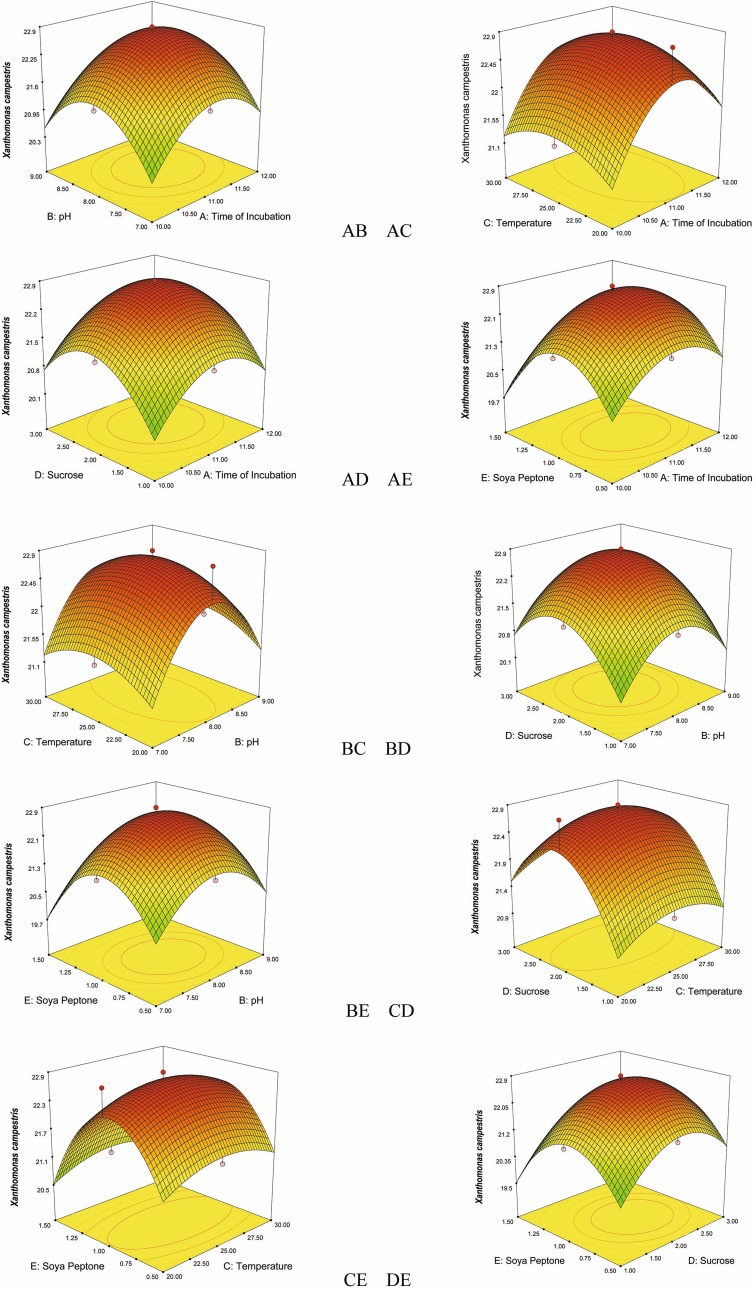
Response surface plots consisting three-dimensional views and contours showing interactive effects of selective variables on zone of inhibition (mm) of the bioactive compound production by VSM 8 against *Xanthomonas campestris:* (*AB*) time of incubation and pH (*AC*) time of incubation and temperature (*AD*) time of incubation and concentration of sucrose, (*AE*) time of incubation and concentration of soya peptone, (*BC*) pH and temperature, (*BD*) pH and concentration of sucrose, (*BE*) pH and concentration of soya peptone, (*CD*) temperature and concentration of sucrose, (*CE*) temperature and concentration of soya peptone, (*DE*) concentration of sucrose and concentration of soya peptone

**Fig. 4:**
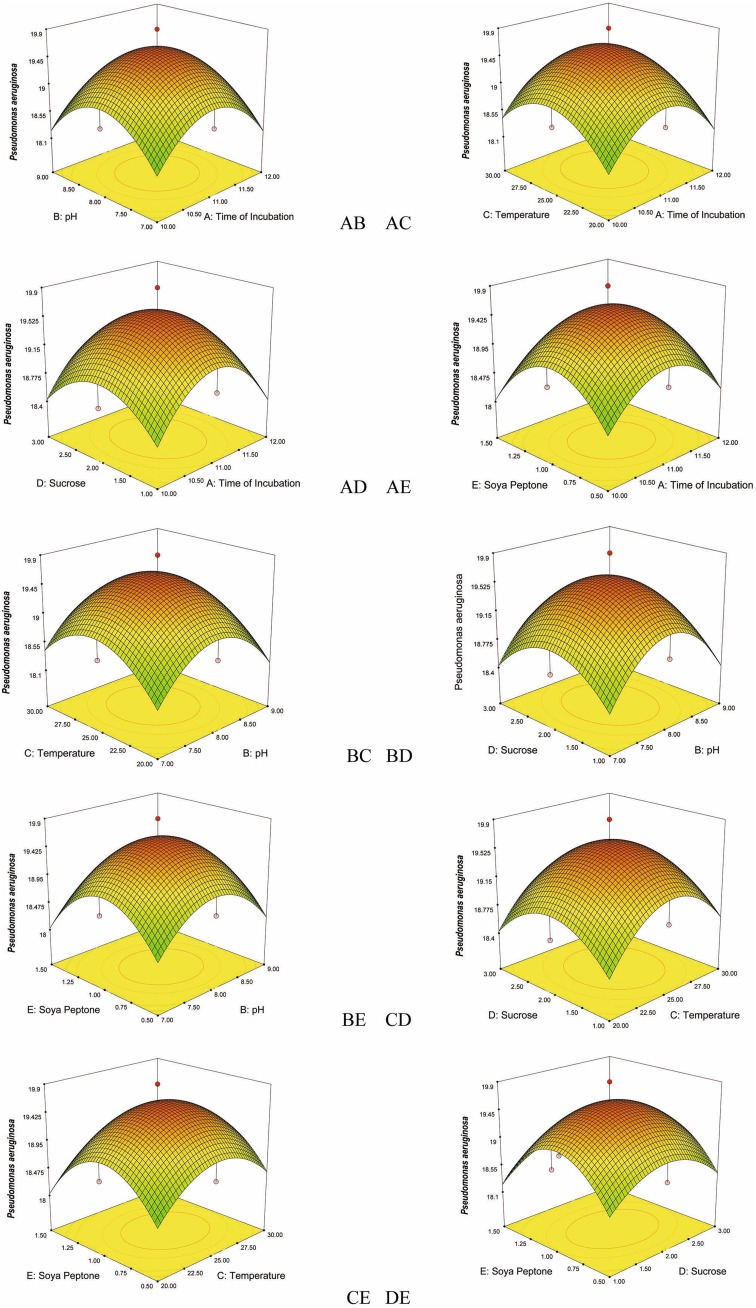
Response surface plots consisting three-dimensional views and contours showing interactive effects of selective variables on zone of inhibition (mm) of the bioactive compound production by VSM 8 against *Pseudomonas aeruginosa*: (*AB*) time of incubation and pH, (*AC*) time of incubation and temperature, (*AD*) time of incubation and concentration of sucrose, (*AE*) time of incubation and concentration of soya peptone, (*BC*) pH and temperature, (*BD*) pH and concentration of sucrose, (*BE*) pH and concentration of soya peptone, (*CD*) temperature and concentration of sucrose, (*CE*) temperature and concentration of soya peptone, (*DE*) concentration of sucrose and concentration of soya peptone

**Fig. 5:**
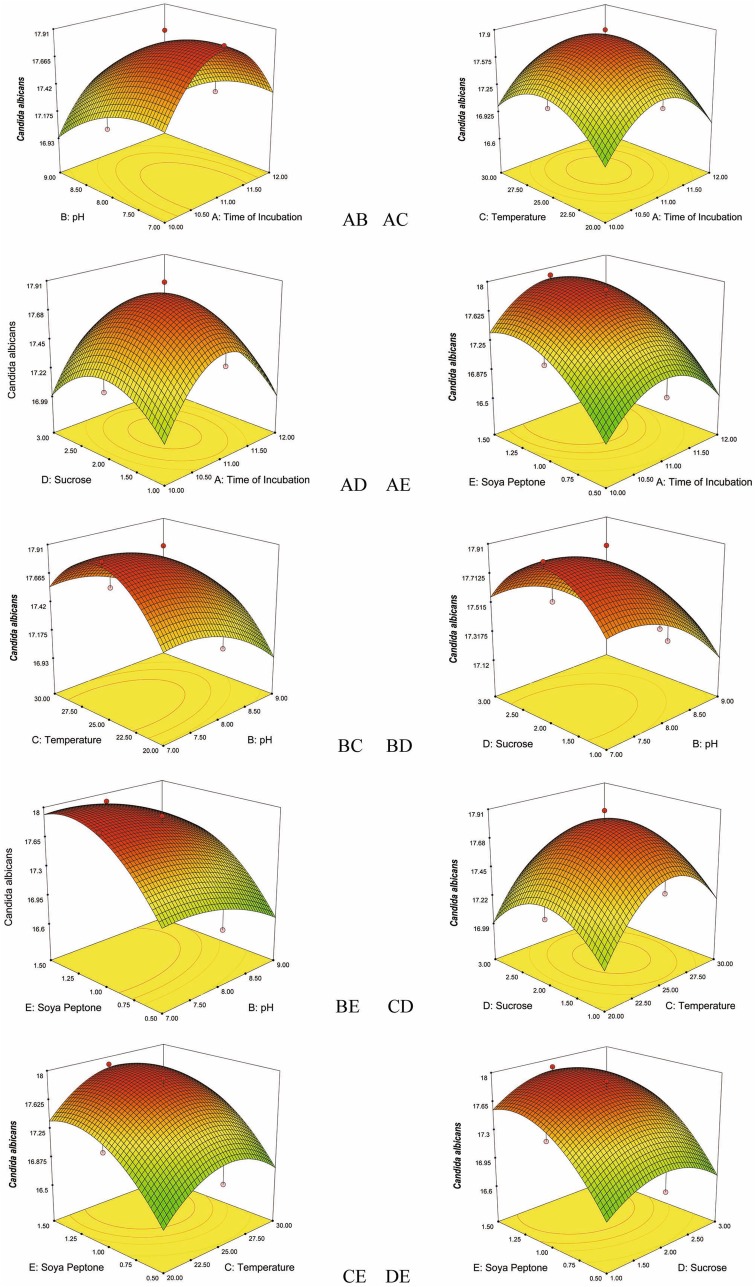
Response surface *plots* consisting three-dimensional views and contours showing interactive effects of selective variables on zone of inhibition (mm) of the bioactive compound production by VSM 8 against *Candida albicans*: (*AB*) time of incubation and pH, (*AC*) time of incubation and temperature, (*AD*) time of incubation and concentration of sucrose, (*AE*) Time of incubation and concentration of soya peptone, (*BC*) pH and temperature, (*BD*) pH and concentration of sucrose, (*BE*) pH and concentration of soya peptone, (*CD*) temperature and concentration of sucrose, (*CE*) temperature and concentration of soya peptone, (*DE*) concentration of sucrose and concentration of soya peptone

The effect of individual parameters, such as time of incubation (in days), pH, temperature, concentration of sucrose, and concentration of soya peptone on the responses, are insignificant, but exhibited significant interactions with other parameters. The significant interactive effects of the variables (incubation time–pH, incubation time-temperature, incubation time-concentration of sucrose, incubation time-concentration of soya peptone, pH–temperature, pH–concentration of sucrose, pH–concentration of soya peptone, temperature–concentration of sucrose, temperature–concentration of soya peptone, and concentration of sucrose–concentration of soya peptone) are represented in 3D plots. The RSM generated 3D plots are used to analyze the effect of the interactions of the variables, and the plots are generated with response on the *z*-axis against two independent variables with third variable kept constant (Figs. 1, 2, 3, 4, 5).

### Effect of the independent variables interactions on the production of bioactive metabolite and its antagonistic effect on the responses

Effect of the different variables with linear square and quadratic coefficients was obtained at significant terms. As single, the factors like concentration of sucrose and soya peptone had high coefficient value which showed their high linear significant effect on growth inhibition of pathogenic microorganisms by the bioactive compound produced by the strain. Direct effect of the five variables, time of incubation, pH, temperature, concentration of sucrose, and concentration of soya peptone on the growth inhibition of the five response pathogens by the bioactive metabolites produced by the strain, is represented by zone of inhibition (mm). The stationary phase of the strain *N. litoralis* strain VSM 8 extended from 5th to 11th day. The maximum yield of the bioactive metabolite was recorded on the 11 day.

Venkata et al. (2011) reported that *Amycolatopsis alba* var. nov. DVR D4 strain produced maximum bioactive compound after 4 days of incubation. However, Abd-Elnaby et al. (2016) reported the maximum production of the bioactive metabolites by *Streptomyces parvus* was obtained after 7 days of incubation. The effect of the environmental factors, such as temperature and pH on the bioactive metabolite production, was studied. When the temperature varied from 20 to 30 °C, the yield of the bioactive metabolite increased gradually and the maximum production was recorded at 25 °C. There was a decrease in the production of the bioactive metabolite with further increase in temperature. Joseph et al. (2009) reported maximum inhibitory activity of the bioactive metabolite produced by marine *Nocardiopsis dassonvillei* MAD08 cultured at 30 °C. Krishna Kumar et al. (2011) also reported the optimum temperature for the bioactive metabolites production by *Streptomyces* sp.—MSU29 as 30 °C. Venkata et al. (2011) reported that the ideal temperature for the production of the maximum bioactive metabolite by *Amycolatopsis alba* var. nov. DVR D4 was 28 °C. Kerstin et al. (2010) reported the marine strain *Nocardiopsis* produced high levels of Thiopeptide Antibiotic, TP-1161 after 14 days of incubation.

The effect of the environmental factors, such as temperature and pH on growth and bioactive metabolite production, was studied. Varied temperatures between 20 and 30 °C were tested for the production of the bioactive compound by *N. litoralis* strain VSM 8. The maximum production of the bioactive metabolite was recorded at 25 °C. These results are in accordance with the results reported by Attiya et al. (2015) who reported the optimum temperature for the production of the bioactive compound by marine *Streptomyces* spp. M19 was 25 °C. Vimal et al. (2009) have reported that the maximum activity of the bioactive metabolite produced by *Nocardiopsis* sp. VITSVK 5 was recorded at 28 °C.

Maximum growth and the elevated level of bioactive metabolite production by *N. litoralis* strain VSM 8 was found to be at pH 8. Increase in pH resulted in the decreased production of the bioactive metabolite. Similar results were reported by Abd-Elnaby et al. (2016) for *Streptomyces parvus*. Kerstin et al. (2010) reported the optimum pH for the production of thiopeptide Antibiotic, TP-1161 by marine strain *Nocardiopsis* was 7.8. Designing and developing the effective media for the bioactive metabolite production are critical, and the impact of carbon needs to be evaluated. Of all the carbon sources tested, significant production of the bioactive metabolites by *N. litoralis* strain VSM 8 was found when the medium was amended with sucrose. The effect of varying concentrations (1–3 %) of the best carbon source selected for the growth and antimicrobial metabolite production was also investigated. The result is in conformity with the results reported by Uddin et al. (2013) for *Streptomyces albolongus*, *Streptomyces* spp. KGG32 (Oskay 2011) and *Streptomyces rochei* G-164 (Chattopadhyay and Sen 1997).

Among the nitrogen sources tested, soya peptone at concentration 1 % was found to influence that maximum production of the bioactive metabolite by *N. litoralis* strain VSM 8. The utilization of the nitrogen source for the production of bioactive metabolites is reported to vary for actinomycetes strains. Singh et al. (2009) recorded that soya bean meal increased antibiotic production by *Streptomyces tanashiensis* strain A2D and similar results were recorded by Narayana and Vijayalakshmi (2008), for *Streptomyces albidoflavus*.

## Discussion

RSM, a collection of statistical and mathematical method, is used in conjunction with central composite design to optimize the different variables at different levels for the production of bioactive metabolite by *N. litoralis* strain VSM 8. Five variables include incubation time, pH, temperature, concentration of sucrose, and soya peptone was optimized by central composite design involving RSM. A high similarity was observed between the predicted and the observed results that reflect the accuracy and accountability of the RSM to optimize the bioactive metabolites production. Of the five variables tested for the correlation between their concentration and production of the bioactive metabolite and its effect against the five responses, all the five variables exhibited significant effect on the production of the bioactive metabolite and their effect against the five pathogens (responses) which is represented as zone of inhibition. Significant interactions between the five variables were observed and analyzed from the 3D surface plots. Application of RSM with CCD statistical experimental design to optimize the selected factors for maximum production is an efficient method that tests the effect of interactions among the variables with minimum number of experiments. Regression equations were derived for both selectivity and total flux using the experimental data together with the statistical software package Design Expert 7.1.3, yielding predicted values in good agreement with observed values.

**Fig. 6:**
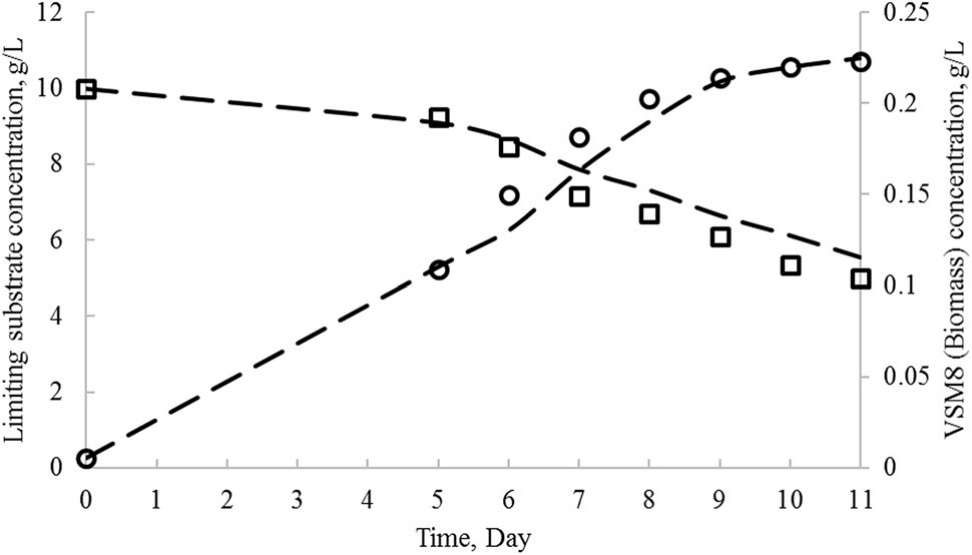
Experimental and model predicted kinetics of biomass growth, substrate utilization. *Open circle*—experimental biomass concentration (g/L), *open square*—experimental substrate concentration (g/L), *dashed lines*—model predicted values (in each case)

**Fig. 7:**
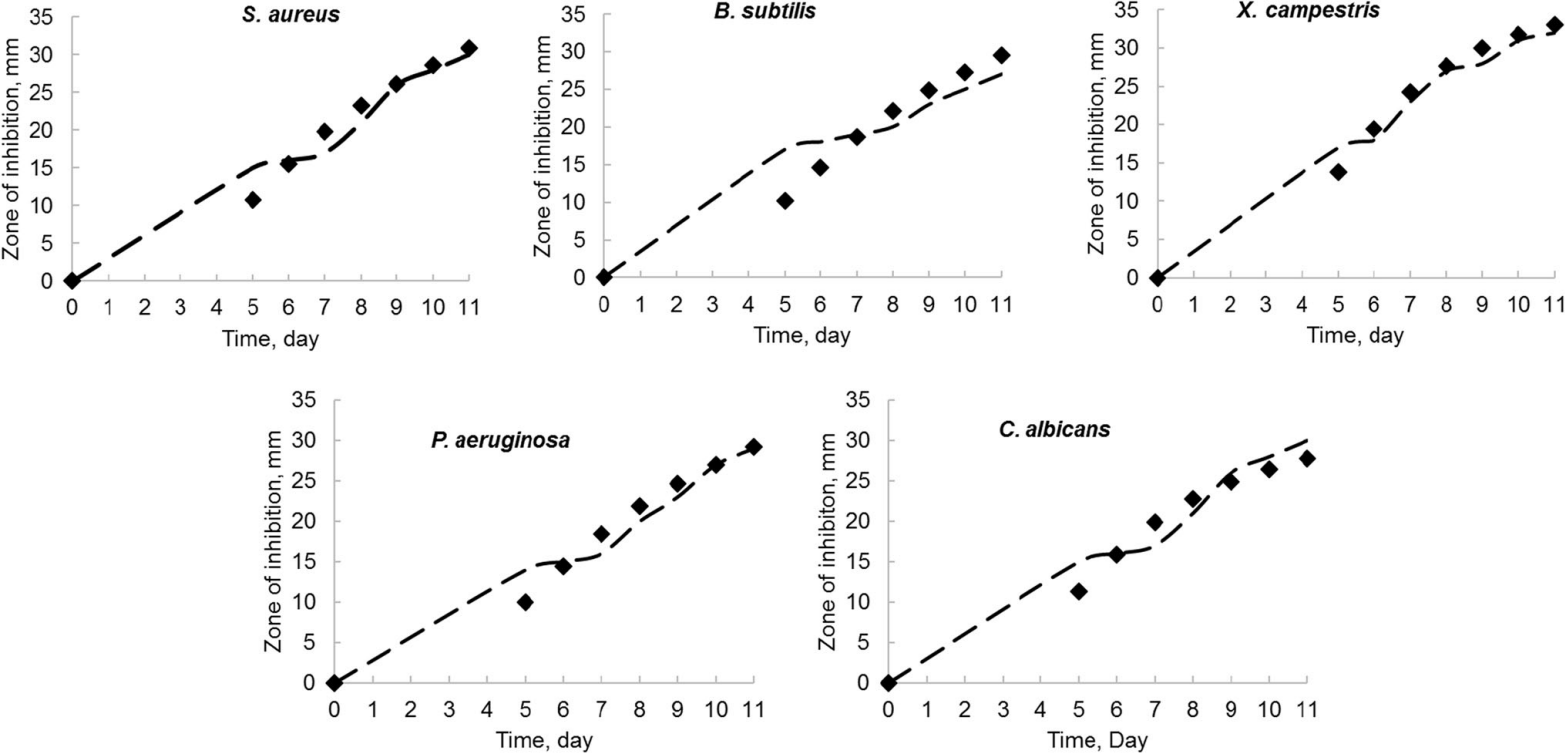
Comparison of experimental and model predicted kinetics of zone of inhibition (mm). *Filled diamond* **—**experimental, *dashed lines*—model

The growth profiles of *N. litoralis* strain VSM 8 (KT901293), limiting substrate utilization results obtained from shake-flask experiments and model kinetics, were compared in Fig. 6. The comparison of experimental versus model predicted zones of inhibition of produced bioactive metabolite on media, inoculated with *S. aureus*, *B. subtilis*, *X. campestris*, *P. aeruginosa*, and *C. albicans* strains over the time, is shown in Fig. 7. From all the profiles, it was observed that model predicted and experimental obtained values show a very good fit. In this study, for fitting of experimental data with unstructured logistic models, non-linear regression using least-square method was done with the help of Microsoft Excel Solver 2010. Biokinetic parameters used in the mathematical model Eqs. (2), (4), and (7) were also estimated and are tabulated in Table 10. The determination coefficient (*R* ^2^) values obtained by fitting logistic (L), logistic incorporated Leudeking–Piret (LILP), and logistic incorporated modified Leudeking–Piret (LIMLP) models to the experimental data were found to be high, thus revealing good precision of the models.

**Table 10:** Estimated kinetic parameters using L, LILP, LIMLP model equations

Kinetic Parameters	*S. aureus*	*B. subtilis*	*X. campestris*	*P. aeruginosa*	*C. albicans*
Logistic (L) model parameters					
* µ*_max_ (day^−1^)	0.7431				
* R* ^2^	0.9683				
* X*_0_ (g/L)	0.005				
* X*_max_ (g/L)	0.226				
Logistics incorporated modified Luedeking–Piret (LIMLP) model parameters					
* γ* (g.S/g.X)	4.8774				
* R* ^*2*^	0.96				
* η* [g.S/(g.X.d)]	2.566371681				
Logistics incorporated Luedeking–Piret (LILP) model parameters					
* α* (mm/g.X)	87.224	81.246	124.62	79.93	100.24
* R* ^*2*^	0.9	0.54	0.9665	0.8877	0.9946
* β* [mm/(g.X.d)]	8.8496	8.8496	4.4248	8.8496	4.4248

From the data of shake flask used in this study, *µ*_max_, *X*_0_ and *X*_max_ were calculated from *N. litoralis* strain VSM 8 (KT901293) growth kinetic profile using logistic (L) model. Values of growth and non-growth-associated product parameters, *α* and *β*, were estimated using LILP model. Higher *α* value than *β* confirmed that bioactive metabolite production by *N. litoralis* strain VSM 8 is more growth-associated than non-growth associated in shake flask. The simulated parameters, *γ* and *η*, of LIMLP model are also in good agreement with the experimental values, which implies that this model is more appropriate to represent limiting substrate utilization kinetics in bioactive metabolite production by *N. litoralis* strain VSM 8. Furthermore, zones of inhibition from agar diffusion tests are much similar to model predicted values (Table 11).

**Table 11:** Comparison of zones of inhibition (mm) from shake-flask experiments and from model

Maximum zone of inhibition (mm)	*S. aureus*	*B. subtilis*	*X. campestris*	*P. aeruginosa*	*C. albicans*
Experimental	30	27	32	29	30
Model fitted	30.81	29.5	33.08	29.21	27.76

## Conclusions

The effect of different variables, including incubation time, pH, temperature, sucrose, and soya peptone concentration on production of bioactive metabolites by *N. litoralis* strain VSM 8 and the inhibiting activity of bioactive metabolites against five pathogens were studied in terms of their responses as inhibition zones (mm). The experimental results showed that the maximum zone of inhibition against all the five responses was found to be 21 mm (*Staphylococcus aureus*), 19.8 mm (*Bacillus subtilis*), 22.9 mm (*Xanthomonas campestris*), 19.9 mm (*Pseudomonas aeruginosa*), and 17.9 mm (*Candida albicans*). The highest production of the bioactive metabolite produced by VSM 8 represented in terms of antimicrobial activity was obtained when the strain was grown in a medium containing 2 % sucrose, 1 % soya peptone with pH 8 incubated at 25 °C for 11 days. However, the range of time of incubation, pH, temperature, concentration of sucrose, and soya peptone above or below the central point reported less zone of inhibition.

A response surface experimental methodology, based on three levels central composite design of experiment, was successfully employed in this optimization study, accounting for the effects of the main variables. The quadratic models developed and subsequent ANOVA test, the concentration of sucrose and soya peptone dosage was found to be the most influential variables for the bioactive metabolite production along with the other significant variables. The model fitted very well to the experimental data, as confirmed by high *R* ^2^ values. RSM with CCD described the production of the bioactive compounds by *N. litoralis* strain VSM 8. Furthermore, the optimum conditions and the effect of bioactive compound against five responses are well induced by 3D plots.

The estimated kinetic parameters for the *N. litoralis* strain VSM 8 growth, and limiting substrate utilization and bioactive metabolite production (in terms of inhibition zones measured against microbial pathogens) showed good regression squares. Thus, the unstructured models provided a better approximation of kinetic profiles of bioactive metabolite production by *N. litoralis* strain VSM 8 in submerged shake-flask fermentations. To the best of our knowledge, this is the first ever report on the kinetic modeling for bioactive metabolite production measured in terms of zones of inhibition by *N. litoralis* strain VSM 8.
